# Protocol for Therapeutic Drug Monitoring Within the Clinical Range Using Mid-infrared Spectroscopy

**DOI:** 10.1021/acs.analchem.4c03864

**Published:** 2024-11-18

**Authors:** Pin Dong, Kezheng Li, David J. Rowe, Thomas F. Krauss, Yue Wang

**Affiliations:** † School of Physics Engineering and Technology, University of York, Heslington, York YO10 5DD, U.K.; ‡ Optoelectronics Research Centre, University of Southampton, Southampton SO17 1BJ, U.K.

## Abstract

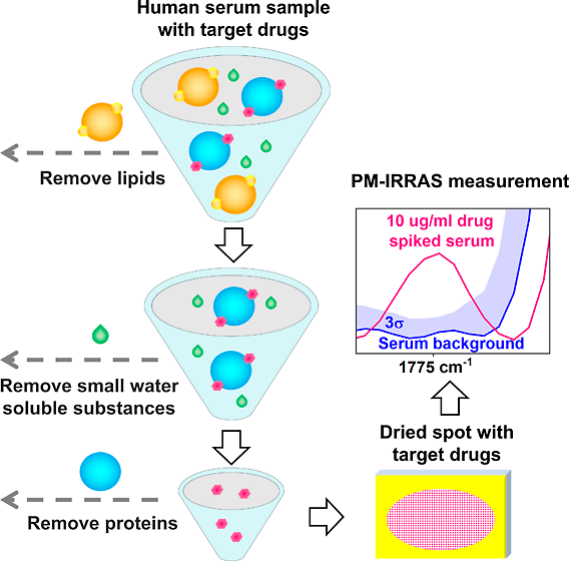

Therapeutic drug monitoring (TDM), which involves measuring drug levels in patients’ body fluids, is an important procedure in clinical practice. However, the analysis technique currently used, i.e. liquid chromatography–tandem mass spectrometry (LC–MS/MS), is laboratory-based, so does not offer the short response time that is often required by clinicians. We suggest that techniques based on Fourier transform infrared spectroscopy (FTIR) offer a promising alternative for TDM. FTIR is rapid, highly specific and can be miniaturized for near-patient applications. The challenge, however, is that FTIR for TDM is limited by the strong mid-IR absorption of endogenous serum constituents. Here, we address this issue and introduce a versatile approach for removing the background of serum lipids, proteins and small water-soluble substances. Using phenytoin, an antiepileptic drug, as an example, we show that our approach enables FTIR to precisely quantify drug molecules in human serum at clinically relevant levels (10 μg/mL), providing an efficient analysis method for TDM. Beyond mid-IR spectroscopy, our study is applicable to other drug sensing techniques that suffer from the large background of serum samples.

Therapeutic drug monitoring (TDM) is an important procedure in clinical practice. By measuring drug levels in patients̀ body fluids, most commonly serum or plasma, TDM is used to monitor and improve treatment outcomes, reduce drug toxicities, avoid the risk of developing drug resistance and optimize personalized drug therapy.^1^ For many drugs, however, the therapeutically effective concentration window (known as “therapeutic range”) can be quite small. For example, phenytoin, a major first-line antiepileptic drug, has a narrow therapeutic range of 10–20 μg/mL, where small dosage adjustments can lead to severe adverse effects, such as seizures and coma.^2^ Similarly, vancomycin, a potent antibiotic for treating methicillin-resistant *Staphylococcus aureus* infections, has a targeted serum concentration of 15–20 μg/mL during a course of treatment, typically ranging from 7 days to several weeks; exceeding this range can result in nephrotoxicity and ototoxicity.^3^ Therefore, monitoring drug levels within the therapeutic range is essential for enhancing clinical efficacy while minimizing toxicities.^1,4^

Currently, liquid chromatography–tandem mass spectrometry (LC–MS/MS) methods are extensively employed in clinical laboratories due to their exceptional selectivity and sensitivity. However, LC–MS is laboratory-based and typically analyses samples in batches, which inevitably incurs several hours̀ delay for routine tests.^5^ In addition, LC–MS is available only in specialized laboratories that are not necessarily accessible to clinicians. Ideally, clinicians would prefer a method that is accurate yet offers a much shorter turnaround time and that allows them to make better informed decisions. Therefore, a reliable assay that can provide results in minutes is highly desired. Commercially available kits based on immunoassays partially address the time requirement, but they often exhibit cross-reactivity and lack of specificity, which limits their utility.^6^ Alternatively, surface-enhanced Raman spectroscopy (SERS) has been proposed for TDM due to its high sensitivity and potential for hand-held devices for bedside measurements.^7,8^ However, as SERS relies on the overlap between the analyte and a nanoscale plasmonic hotspot, reproducibility and quantification are challenging.^9^ We suggest that Fourier transform infrared (FTIR) spectroscopy provides a useful compromise between these methods. Similar to Raman, it directly measures molecular vibrations and generates a unique spectrochemical fingerprint but it also allows extraction of both qualitative and quantitative information.^10^ Furthermore, FTIR-based techniques hold the potential to be used as miniaturized instruments.^11^ Among the various FTIR techniques, attenuated total reflection (ATR)-FTIR stands out for its high sensitivity, user-friendly operation and rapid data acquisition.^10^ These attributes position ATR-FTIR as a promising TDM technique with the potential to streamline clinical decision-making processes.

The presence of water and proteins in human serum, however, limits the application of ATR-FTIR in TDM, as the absorption peaks of proteins in the range of 1700–1400 cm^–1^ overlap with the fingerprint region of most drugs.^12^ The absorption peaks of proteins typically have a much higher magnitude than drugs at therapeutic concentrations, which makes it difficult to isolate the fingerprint of most drugs from proteins using spectral postprocessing. Water is problematic as a matrix because its high absorption reduces the dynamic range and sensitivity of any absorption-based characterization method in this wavenumber range. Additionally, other endogenous substances such as sugars, lipids and peptides contribute to this background, making the quantitative analysis of small drug molecules even more challenging.^13^ Studies have demonstrated the necessity of removing water and proteins to improve the limit of detection (LOD) of FTIR techniques.^13−16^ For example, Wood et al.^15^ showed that by removing water through drying, ATR-FTIR could quantify glucose in the blood at a concentration of 300 μg/mL. Further removal of albumin by centrifugal filtration allowed ATR-FTIR to determine glucose in human serum at a 10-fold lower concentration of 30 μg/mL.^14^ It should be noted, however, that centrifugal filtration also leads to significant drug loss, especially for highly protein-bound drugs. For example, in human serum, more than 90% of phenytoin and warfarin, both on the TDM list,^17^ are bound to albumin.^4,18^ So the additional challenge is to remove water and proteins with minimal drug loss.

In addition, even after removing the protein and water background using current methods, the LOD of ATR-FTIR is still not sufficient to assess many drugs that require clinical monitoring. This restriction is due to other endogenous serum substances, such as lipids and metabolites, that can interact with the drug of interest, thereby contributing to background noise and limiting the LOD. Therefore, there is a clear need for a new sample preparation method for human serum that removes most or all of this background and thereby enables the use of FTIR-based techniques in TDM. The method needs to remove proteins, lipids and other metabolites and be compatible with drying to remove the water background, while maintaining a relatively low drug loss.

Here, we introduce a new approach that meets these requirements. We demonstrate that our approach significantly improves the LOD of FTIR-based techniques by markedly reducing the serum background. As a result, we are able to demonstrate that FTIR spectroscopy is able to quantify drugs in human serum at clinically relevant levels.

## Experimental Section

### Material

Phenytoin (5,5-diphenylhydantoin, purity ≥98%) was obtained from Cayman Chemical. 4-Hydroxybenzonitrile (purity ≥98%) was purchased from Thermo Scientific Chemicals. Ethyl acetate (anhydrous, 99.8%) for liquid–liquid extraction, dextran sulfate sodium salt (*M*_r_ ≈ 40,000), and ammonium sulfate (NH_4_)_2_SO_4_ (≥99.0%) for albumin precipitation were purchased from Merck Life Science Limited. All other chemicals and reagents were of analytical grade. HPLC-grade acetonitrile was supplied by Fisher Chemical (Loughborough, United Kingdom). Magnesium chloride hexahydrate (≥99.0%) was obtained from Fluorochem Ltd., UK. A mixture of MgCl_2_ (3 mol/L) and dextran sulfate sodium (6%) solution was used for lipoprotein precipitation. Blank human serum (male AB, USA origin, sterile-filtered) was acquired from Merck Life Science Limited and stored at −20 °C until analysis.

## Methods

### FTIR-Based Measurements

#### ATR-FTIR

A Fourier transform infrared spectrometer (VERTEX 70, Bruker) with a DTGS (deuterated triglycine sulfate) temperature-stabilized coated detector was used. The instrument was equipped with a three-bounce ATR accessory with a diamond/ZnSe crystal (MIRACLE, PIKE Technologies). Samples measured by ATR-FTIR are phenytoin dissolved in ethyl acetate. A drop of 10 μL of phenytoin ethyl acetate samples was first dried on the ATR crystal and then measured after taking the atmosphere as the background spectrum.

#### PM-IRRAS

Measurements were performed using the same VERTEX 70 Fourier transform infrared spectrometer with an angle of incidence of 80° relative to the substrate surface normal. For the *p*-polarization of the IR light, an aluminum wire grid was used and modulated at 50 kHz with a ZnSe photoelastic modulator (PEM, Bruker PMA-50). Light reflected from the sample was focused with a ZnSe lens onto a cryogenic mercury cadmium telluride (MCT) detector. The optical path of the spectrometer was purged with dried air. All the infrared spectra were collected using OPUS software (Bruker) with a spectral resolution of 4 cm^–1^ and the accumulation of 100 scans in the range of 4000–400 cm^–1^. The data analysis was performed by Origin software.

### HPLC

The HPLC system was an Agilent 1290 Infinity LC system with a photodiode array detector set to 220 nm (Agilent Technologies, USA). Chromatographic separation was carried out on a ZORBAX SB-C18 Column (4.6 × 150 mm, 5 μm, Agilent) and protected by SB-C18 Guard Cartridges (4.6 × 12.5 mm, 5 μm, Agilent). The column oven temperature was set at 20 °C. An isocratic mobile phase consisted of 35% acetonitrile and 65% sodium acetate (0.02 M, adjusted to pH 4.6 with acetic acid). The flow rate of the mobile phase and injection volume were 1.0 mL/min and 5 μL, respectively. The total run time was 12 min. A set of five samples, each of them in triplicate, containing 5, 10, 20, 30, 40 μg/mL of phenytoin was prepared in acetonitrile and was used as the calibration set (*R*^2^ > 0.999).

### Preparation of Calibration Standards and Quality Control Samples

The stock solution was prepared by dissolving accurately weighed amounts of phenytoin in methanol to yield a 10 mg/mL drug concentration. The working solutions (150, 300, 450, 600, and 750 μg/mL) were prepared by further dilution of stock solution with methanol. The internal standard containing 1500 μg/mL 4-hydroxybenzonitrile was prepared in methanol.

The calibration standards were prepared by adding 10 μL of the working solutions to 300 μL of blank serum aliquots, yielding final serum drug concentrations of 5, 10, 15, 20, and 25 μg/mL. Quality control samples were prepared independently at two concentration levels: one within and one above the therapeutic window of phenytoin (10–20 μg/mL). These samples were analyzed by both HPLC and PM-IRRAS. Each drug spiked serum sample was processed as described in the sample preparation section. Six replicates were analyzed for each calibration standard and quality control sample.

### Serum Sample Preparation

The phenytoin-spiked serum samples (300 μL) were mixed with 10 μL of the 4-hydroxybenzonitrile methanol solution with a concentration of 1500 μg/mL (internal standard) with the vortex for 1 min at 3000 rpm, resulting in a final internal standard concentration of 50 μg/mL. Next, 10 μL of a solution containing 3 mol/L magnesium chloride and 6% (w/v) of dextran sulfate sodium salt was added, yielding final concentrations of 0.1 mol/L magnesium chloride and 0.2% (w/v) dextran sulfate sodium salt. The mixture was vortexed again for 1 min at 3000 rpm and centrifuged at 10,000 rpm (Eppendorf MiniSpin) for 3 min. The supernatant was transferred to 2 mL centrifuge tubes. Subsequently, 1200 μL of saturated ammonium sulfate was added to the supernatant, reaching a final concentration of 80% saturated ammonium sulfate. The mixture was vortexed for 1 min at 3000 rpm and centrifuged for 2 min at 10,000 rpm. The upper layer was discarded, and the precipitates were resuspended in 300 μL of distilled water. Next, 1 mL of ethyl acetate was added to each sample, followed by the vortex for 2 min at 3000 rpm and centrifugation at 10,000 rpm for 2 min. The organic (upper) layer was transferred to 2 mL centrifuge tubes. The extraction process was repeated, and the upper layers from the two extractions were combined and evaporated under constant airflow at room temperature. The resulting dry residues were redissolved in 300 μL of either acetonitrile for HPLC analysis or ethyl acetate for PM-IRRAS measurements with vortexing for 2 min at 3000 rpm. For PM- IRRAS measurements, 200 μL of the ethyl acetate sample was naturally dried at room temperature on a gold-coated silicon substrate with a size of 25 mm × 25 mm. The dried spot is not uniform, which may lead to increased uncertainty, however, this has been taken into consideration by spatial averaging, i.e. taking the averaged PM-IRRAS spectrum from four measurements with rotation of the substrate, see Figures S4 and S5. Drug loss was measured using HPLC and calculated according to the equation , where *m*_add_ is the amount of drug added to the serum sample and *m*_collect_ are is the amount collected after sample preparation.

## Results and Discussion

### Verification of the FTIR-Based Techniques for TDM

**Figure 1:**
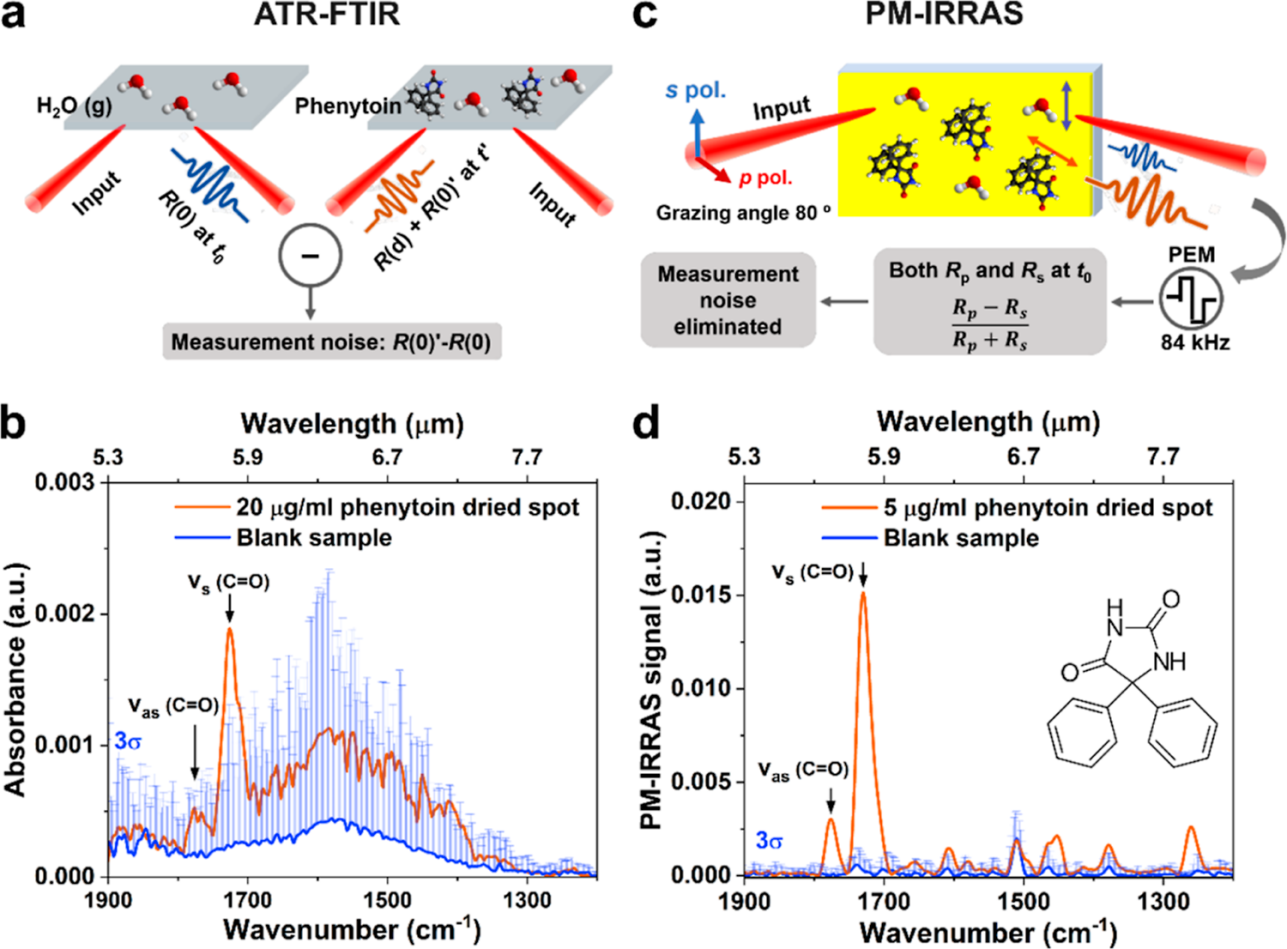
Two FTIR techniques, ATR and PM-IRRAS, to measure dried phenytoin spots. Schematic of the principles of ATR-FTIR (a) and PM-IRRAS (c); (b) ATR-FTIR spectrum measured from a dried spot of phenytoin dissolved in ethyl acetate at a concentration of 20 μg/mL (orange line) and a blank sample (blue line). The two peaks at 1772 and 1726 cm^–1^ (highlighted by arrows) correspond to the asymmetric and symmetric stretching of the carbonyl group of the molecule, respectively; (d) PM-IRRAS spectrum measured from of a dried spot of phenytoin dissolved in ethyl acetate at a concentration of 5 μg/mL and a blank sample. The inset shows the chemical structure of phenytoin. The blue shadings in (b) and (d) represent the measurement noise calculated as the 3-fold standard deviation (3σ) of ten repeated measurements of blank samples. This noise primarily includes atmospheric noise from water vapor and carbon dioxide.

We first investigated the LOD of ATR-FTIR for the chosen model drug, phenytoin (Figure 1a,b). Initially, we used phenytoin dissolved in ethyl acetate, followed by drying, to study the influence of water removal while excluding other factors. Figure 1b shows the ATR-FTIR spectrum of a dried spot of phenytoin at a concentration of 20 μg/mL. The two peaks at 1772 and 1726 cm^–1^ correspond to the asymmetric and symmetric stretching, respectively, of the carbonyl group of the molecule. The blue shading represents the 3-fold standard deviation (3σ) of the instrumental and atmospheric noise, the latter primarily caused by water vapor and carbon dioxide, calculated from 10 repeated blank measurements. We note that the two phenytoin peaks are nearly buried in the noise, indicating that the LOD of ATR-FTIR is not sufficient to meet the requirement for quantifying clinical samples with drug levels below 20 μg/mL. For example, the 3σ at 1772 cm^–1^ is 6 × 10^–5^, equivalent to a calculated LOD of 28 μg/mL.

To reduce the noise and improve the LOD, we pivoted to the method of polarization-modulation infrared reflection–absorption spectroscopy (PM-IRRAS). This technique is widely used in surface chemistry for characterizing monolayers and thin films.^19,20^ PM-IRRAS minimizes atmospheric interference by simultaneously measuring both the sample and atmospheric background using *p*- and *s*-polarized light (Figure 1c).^21−24^ Despite its enhanced sensitivity, PM-IRRAS has been rarely utilized in TDM because of the strong background of water, proteins, lipids and other small molecules. In this study, we explored the feasibility of using PM-IRRAS in TDM. As shown in Figure 1d, the improved spectral signal together with the reduced impact of atmospheric noise demonstrates that PM-IRRAS can minimize the interference of water vapor. We note that the peaks at 1772 and 1726 cm^–1^ of a 5 μg/mL phenytoin dry spot (below the clinical range) are now well above the noise level. The 3σ at 1772 cm^–1^ is equivalent to a LOD of 0.5 μg/mL, showing that PM-IRRAS can meet the quantification requirement for clinical samples. Consequently, we used PM-IRRAS for all subsequent investigations.

### Protein Removal

**Figure 2:**
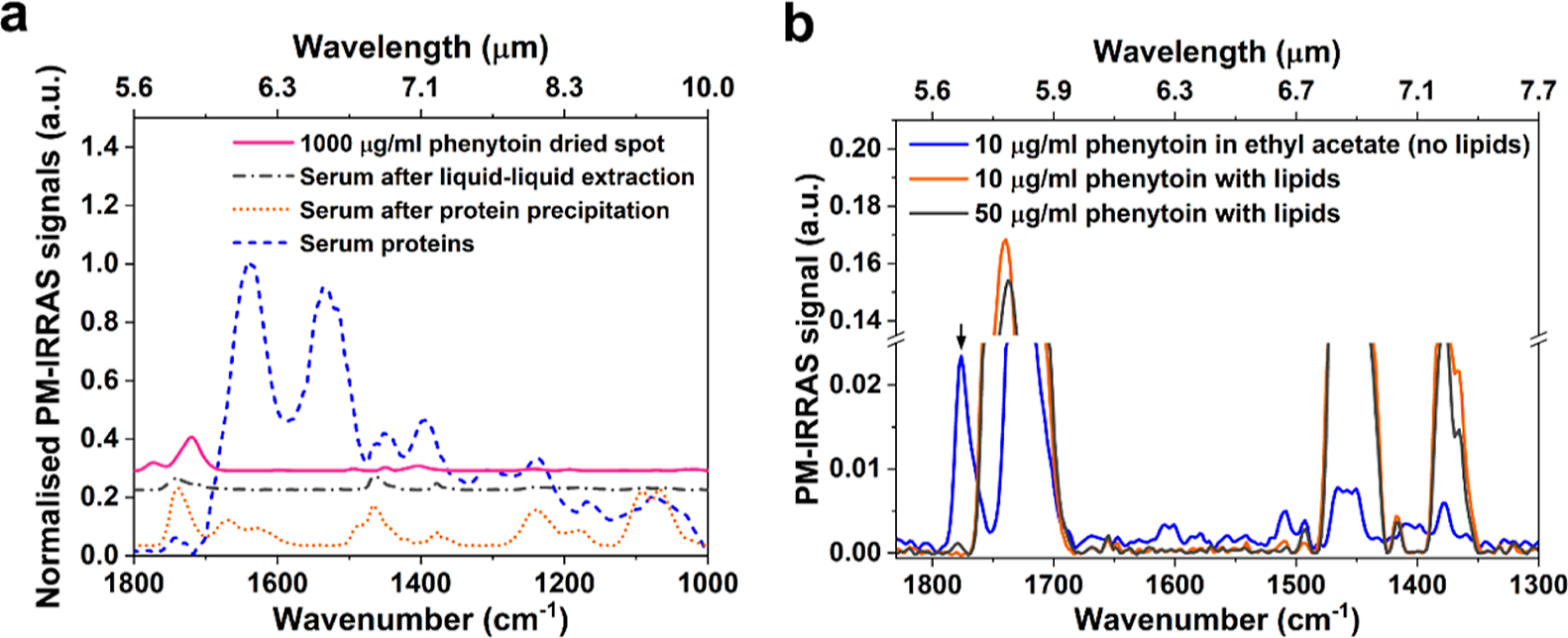
(a) PM-IRRAS spectra following protein removal by protein precipitation (dotted line) and liquid–liquid extraction (dash-dotted line), compared to dried serum proteins (dashed line) and pure phenytoin (solid line); The *y*-axis values are offset for clarity. (b) PM-IRRAS spectra of dried spots of phenytoin with and without serum lipids at concentrations of 10 and 50 μg/mL. With the presence of serum lipids (orange and blue lines), the 1772 cm^–1^ target peak is significantly diminished.

Having discussed the need for serum protein removal without causing excessive drug loss, we now investigate the possible methodologies in greater depth. There are two main methods: protein precipitation and liquid–liquid extraction. Protein precipitation, employing solvents such as acetonitrile and methanol, is commonly used in HPLC-MS analysis.^25^ For example, acetonitrile can effectively eliminate 97% of serum proteins while extracting the majority of the target drug molecules.^26^ When we applied the protein precipitation method to our serum samples, however, we only saw limited success. For example, we observed a significant remaining signature of the amide I (1700–1600 cm^–1^) and II (1600–1500 cm^–1^) bands as shown in Figure 2a, which likely originate from peptides of low molecular weight present in serum. Furthermore, we observed strong absorption at 1740, 1236 and 1090 cm^–1^, which we attribute to the stretching of C=C for neutral lipids of triglycerides, as well as the C=O and PO^2–^ groups of phospholipids.^27,28^

We then considered the liquid–liquid extraction method, which utilizes a water-immiscible solvent such as ethyl acetate or chloroform to extract lipophilic drugs into the solvent while depleting serum proteins.^29^ The result is shown in Figure 2a. We note that ethyl acetate has already been employed in several studies for extracting phenytoin from human plasma.^30−32^ We observe that, in contrast to protein precipitation, the serum background extracted by ethyl acetate exhibits no amide bands, indicating efficient removal of serum proteins. The intensity of the characteristic peak of 1740 cm^–1^ is also decreased by a factor of 5, suggesting a reduced amount of other serum substances. Therefore, it is clear that liquid–liquid extraction with ethyl acetate is the preferred method for removing serum proteins and water. The downside of the liquid–liquid extraction is that serum lipids are extracted together with the phenytoin due to the lack of specificity of ethyl acetate to the drug. This is apparent from Figure 2b, where we observe a number of absorption peaks (1740, 1465 and 1376 cm^–1^) that are associated with lipids^27,28^ and that interfere with the phenytoin peaks. The exception is the peak at 1772 cm^–1^ (highlighted by an arrow). This peak corresponds to the asymmetric stretching of the carbonyl group of the hydantoin^33^ and because it exhibits the lowest background, we select it as the target peak for phenytoin quantification.

### Lipid Removal

As mentioned above, the liquid–liquid extraction method also extracts some nonpolar substances, such as serum lipids, together with phenytoin. We note that serum lipids interfere with the 1772 cm^–1^ drug peak. For example, at a drug concentration of 10 μg/mL, as shown in Figure 2b, the target peak is barely discernible with serum lipids present, and we need to increase the drug concentration to 50 μg/mL to allow the peak to stand out clearly. For reference, we show the phenytoin peak at 10 μg/mL in ethyl acetate; the peak is very clear without lipids. This reduction is due to the molecular interaction (e.g., hydrogen bond) between serum lipids and phenytoin, which broadens the target peak, thereby reducing its intensity to a level that is too low to observe.^34^ This observation highlights the importance of removing serum lipids to improve the LOD.

**Figure 3:**
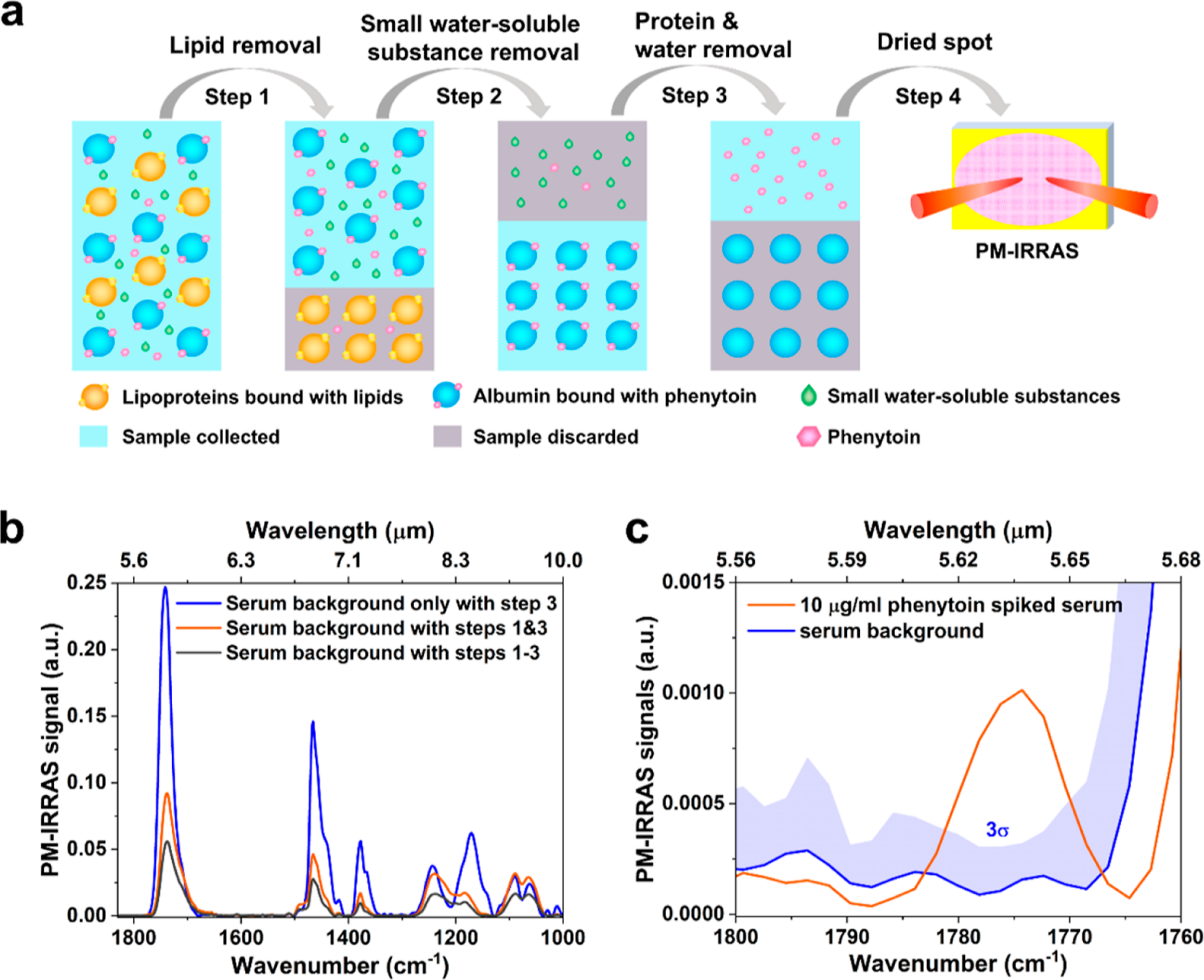
Protocol for human serum sample preparation to remove lipids, small water-soluble substances and proteins and water. (a) Schematic diagram of the protocol. Step 1, lipid removal: a mixture of magnesium chloride (3 mol/L)-dextran sulfate sodium (6%)^37^ is added to precipitate lipoproteins, to which serum lipids such as triglycerides and cholesterol bind. Step 2, removal of small water-soluble substances: saturated ammonium sulfate is added to precipitate hydrophilic proteins such as albumin, to which the target drug phenytoin binds. The supernatant, involving small water-soluble molecules, is subsequently discarded. Step 3, protein removal: the water-immiscible solvent ethyl acetate is used to extract phenytoin while removing serum proteins. Step 4, dried spot: the upper solvent layer is collected and dried on a gold-coated silicon substrate for PM-IRRAS measurements; (b) PM-IRRAS spectra highlighting the successive reduction of the serum background after the various steps used in the protocol; (c) A zoomed-in spectrum of the 1772 cm^–1^ peak highlighting the successful conclusion of the protocol. A concentration of 10 μg/mL is clearly identifiable, well above the 3σ of serum background, calculated from six replicates (indicated by blue shading).

Cholesterol and triglycerides are the predominant types of serum lipids, most of which are bound as lipoproteins.^35^ Lipids are commonly extracted with a chloroform–methanol mixture.^36^ However, using chloroform–methanol for lipid removal will lead to a significant drug loss as phenytoin is soluble in these solvents. Instead, a combination of magnesium chloride and dextran sulfate sodium has been utilized previously to selectively precipitate lipoproteins by forming insoluble complexes through electrostatic interactions and charge neutralization.^37^ Consequently, we used this mixture to remove serum lipids, with the protocol shown in Figure 3a and the results in Figure 3b,c. In Figure 3b, we highlight the characteristic lipid peak at 1740 cm^–1^, which decreases approximately 3-fold following lipoprotein precipitation, indicating the successful removal of serum lipids.

### Small Water-Soluble Substances Removal

The removal of both lipids and proteins enables PM-IRRAS to detect the lowest drug concentration of 15 μg/mL in serum (Figure S1). Unfortunately, there is still too much serum background to achieve an LOD value below 10 μg/mL. We suggest that the remaining background arises from the presence of residual small water-soluble substances such as amino acids, hormones, and acetoacetate, which continue to interact with phenytoin and attenuate the target peak.^38,39^ Thus, further removal of small water-soluble substances from the serum is necessary to improve the LOD of PM-IRRAS.

To achieve this, we used saturated ammonium sulfate to precipitate the albumin following lipid removal. High concentrations of ammonium sulfate (60–80% saturation) cause most serum proteins to precipitate through a process known as “salting out,” which exploits the decreased solubility of proteins in high-salt environments.^40^ Since more than 90% phenytoin binds to albumin, we collected the albumin precipitates and discarded the supernatant that contains most of the small water-soluble substances. As shown in Figure 3b, further removal of the small water-soluble substances reduced the serum background by an additional factor of 1.5, taking the peak intensity at 1740 cm^–1^ as a reference. Lastly, the target drug molecules were extracted from the albumin precipitates. This refinement led to a further improvement in the LOD of PM-IRRAS. As shown in Figure 3c, the target peak significantly surpassed the 3σ threshold of the background signals, allowing PM-IRRAS to quantify phenytoin in serum concentration as low as 10 μg/mL. The total drug loss associated with the removal of lipids in the first step and small water-soluble substances in the second step is only 30% (Figure S2). Meanwhile the reduction in serum background is approximately a factor of 7.5. Consequently, the protocol significantly increases the signal-to-noise ratio overall.

### Absolute Quantification of Phenytoin in Human Serum

**Figure 4:**
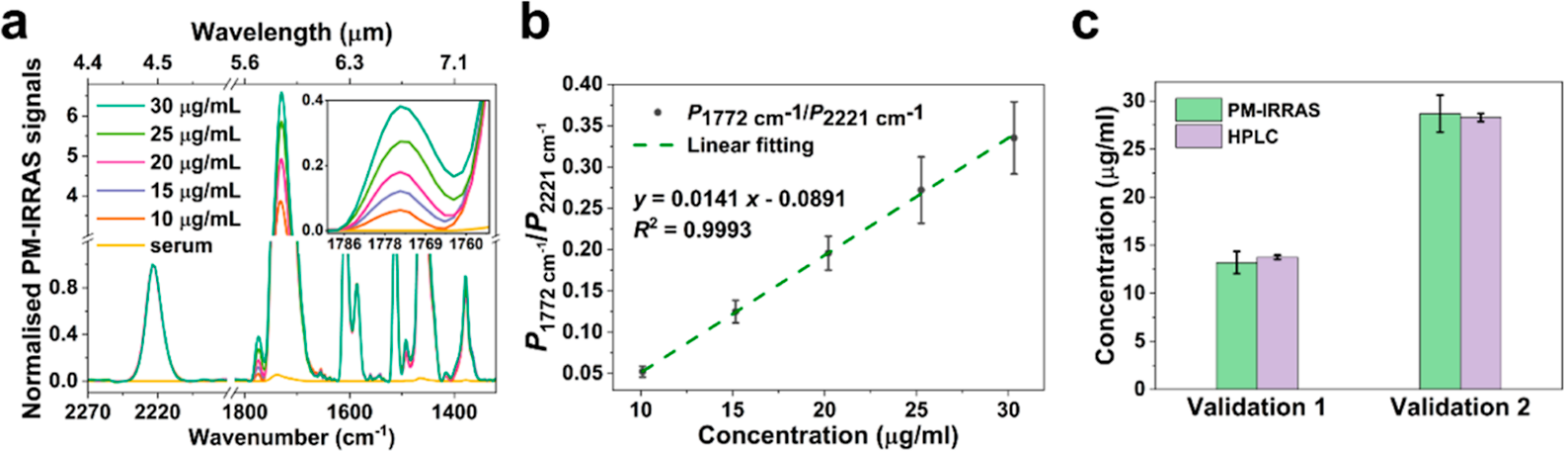
Calibration curve of PM-IRRAS FTIR and validation of serum samples. (a) PM-IRRAS FTIR spectra of phenytoin spiked samples at concentrations of 10, 15, 20, 25, 30 μg/mL, along with blank serum, following preparation with our new protocol. Each spectrum is normalized to the peak height of the internal standard at 2221 cm^–1^. Inset: a zoom-in spectrum of the target peak at 1772 cm^–1^; (b) calibration curve of phenytoin spiked serum measured via PM-IRRAS. Six replicates are measured for each concentration; (c) validation measurements of PM-IRRAS FTIR compared to HPLC at two concentrations within the range shown in (b). Each concentration was analyzed in six replicates.

Finally, we applied our new protocol to a series of phenytoin-spiked serum samples. We added an internal standard to aid quantification, i.e. 4-hydroxybenzonitrile. This molecule exhibits a sharp C≡N stretching peak at 2221 cm^–1^, where there is no serum background; the molecule also does not interfere with phenytoin (Figure 4). When increasing the drug concentration in the clinical range of 10–30 μg/mL, we observe that the 1772 cm^–1^ target peak increases proportionally, as shown in Figure 4a. The ratio of the peak height at 1772 and 2221 cm^–1^ can then be used to establish the calibration curve. Figure 4b highlights the excellent linearity between the PM-IRRAS signals and the drug serum concentrations, with a correlation coefficient of 0.9993. We used HPLC as the validation method (Figure S3). For quality control samples, we used two concentrations that represent the range of quantification in Figure 4a, i.e. 13.2 and 28.7 μg/mL, respectively, and we also note excellent agreement. Nevertheless, we note that the standard deviation of the PM-IRRAS measurements is larger than that of the HPLC analysis. We explain this larger deviation with the homogeneity issue of dry spots, which we discuss in more detail in the Supporting Information (Figures S4 and S5). Overall, Figure 4 provides clear evidence that PM-IRRAS, in conjunction with the multistage protocol we introduce here, is capable of quantifying phenytoin in serum samples at the clinically relevant range.

Several aspects can be further investigated in future studies. First, we measured the total serum concentration of the drug of interest based on the sample preparation approach rather than the free drugs, which are more directly related to efficacy. Nevertheless, the second step of our protocol of removing small water-soluble substances already separates free drugs, offering the possibility of quantifying free drug molecules in serum. Second, while the current approach is applicable to highly protein-bound drugs with poor solubility in water, it requires further adjustments for water-soluble drugs, based on the same principle of reducing most of the serum endogenous constituents. For example, vancomycin, typically less protein-bound, could benefit from nanoparticle absorption methods to selectively collect water-soluble drug molecules while removing other endogenous substances.^41^ Lastly, the current protocol is entirely manual. An essential next step toward realizing a true near-patient test is to simplify and automate this process. To this end, we note that other studies have already demonstrated the ability to separately remove lipoproteins^42^ and albumin proteins^43^ using microfluidic circuits. Therefore, integrating our protocol into a microfluidic platform is feasible and could potentially provide a building block toward TDM in clinical settings using mid-IR spectroscopy.

## Conclusions

In this work, we introduced a practical and widely applicable approach for human serum sample preparation, enabling an FTIR spectroscopy-based technique to quantify drug serum concentrations at clinically relevant levels. Our study demonstrates the many advantages of FTIR-based techniques for TDM. It emphasizes the importance of removing serum lipids, small water-soluble substances and proteins to achieve the required performance. While our protocol is verified with phenytoin, it is broadly applicable to other poorly water-soluble and highly protein-bound drugs, such as warfarin, tacrolimus, and digitoxin, commonly monitored in clinical settings.^4^ Beyond FTIR-based techniques, our serum preparation protocol can also be applied to improve the LOD of Raman-based techniques such as SERS, as protein and lipid background interreferences are major concerns for these techniques as well. Additionally, the approach opens up the utility of mid-IR based resonant sensing modalities to serum-based TDM, which have previously been limited to laboratory media-based samples.^44−47^ Besides its high sensitivity and specificity, our method reduces the total time from serum sample preparation to the final drug concentration measurement to as little as 20 min. This demonstrates significant potential for optimizing clinical workflows, enabling more efficient decision-making and improved treatment outcomes. The total cost of the materials used in the protocol is approximately $0.4, highlighting the cost-effectiveness of the method (Table S1).

In conclusion, we offer a versatile solution for serum sample preparation that markedly enhances the performance of mid-infrared spectroscopy for TDM. Importantly, our protocol is also transferable to other sensing technologies. Overall, our work contributes to the introduction of a miniaturized on-site mid-IR sensing modality aimed at improving patients’ quality of care.

## Supporting Information Available

PM-IRRAS FTIR spectra of phenytoin-spiked human serum following different sample preparation steps; drug loss of phenytoin at each step of the serum sample preparation, measured by HPLC; the calibration curve established with an internal standard; PM-IRRAS FTIR measurements with spatial averaging; the relative standard deviations of the serum sample preparation protocol, and costs of materials used in the established serum sample protocol (PDF)
