# Supplementary material for: Protocol for Therapeutic Drug Monitoring Within the Clinical Range Using Mid-infrared Spectroscopy
Source: Anal Chem. 2024 Nov 18;96(48):19021–8. doi: 10.1021/acs.analchem.4c03864 (PMC11618749; doi:10.1021/acs.analchem.4c03864)
Supplement: Supplementary file 1 — ac4c03864_si_001.pdf [file ac4c03864_si_001.pdf]

Supporting Information

Protocol for Therapeutic Drug Monitoring Within the Clinical Range Using Mid-Infrared Spectroscopy

Pin Dong[1*], Kezheng Li[1], David J. Rowe[2], Thomas F. Krauss[1*] and Yue Wang[1*]

1. School of Physics Engineering and Technology, University of York, Heslington, York YO10 5DD, UK

2. Optoelectronics Research Centre, University of Southampton, Southampton, SO17 1BJ, UK

*Corresponding Authors:

Pin Dong; Email: pin.dong@york.ac.uk;

Thomas F. Krauss; Email: thomas.krauss@york.ac.uk;

Yue Wang; Email: yue.wang@york.ac.uk.

Contents

Figure S1. PM-IRRAS FTIR spectra of phenytoin-spiked human serum following different sample preparation steps. S3

Figure S2. Drug loss of phenytoin at each step of the serum sample preparation, measured by HPLC. S3

Figure S3. The calibration curve established with an internal standard. S4

Figure S4. PM-IRRAS FTIR measurements with spatial averaging. S4

Figure S5. The relative standard deviations of the serum sample preparation protocol. S5

Table 1 Costs of materials used in the established serum sample protocol......S5

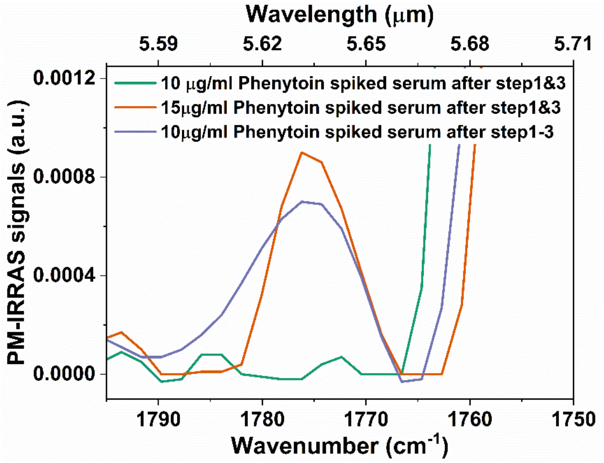

Figure S1. PM-IRRAS FTIR spectra of phenytoin-spiked human serum following different sample preparation steps. The green and orange lines represent the drug-spiked serum samples after step 1 (lipid removal) and step 3 (protein and water removal by liquid-liquid extraction), respectively, while the purple line represents the serum sample prepared using the complete three-step protocol (lipid removal, small water-soluble substances removal, and protein removal).

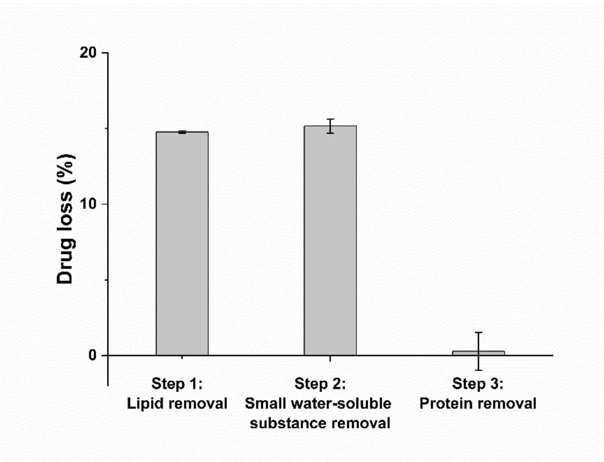

Figure S2. Drug loss of phenytoin at each step of the serum sample preparation, measured by HPLC. Both steps 1 and 2 result in an approximately 15% drug loss at each stage. The final step of liquid-liquid extraction incurs minimal drug loss (0.3%). The total drug loss of the protocol is therefore ≈30%.

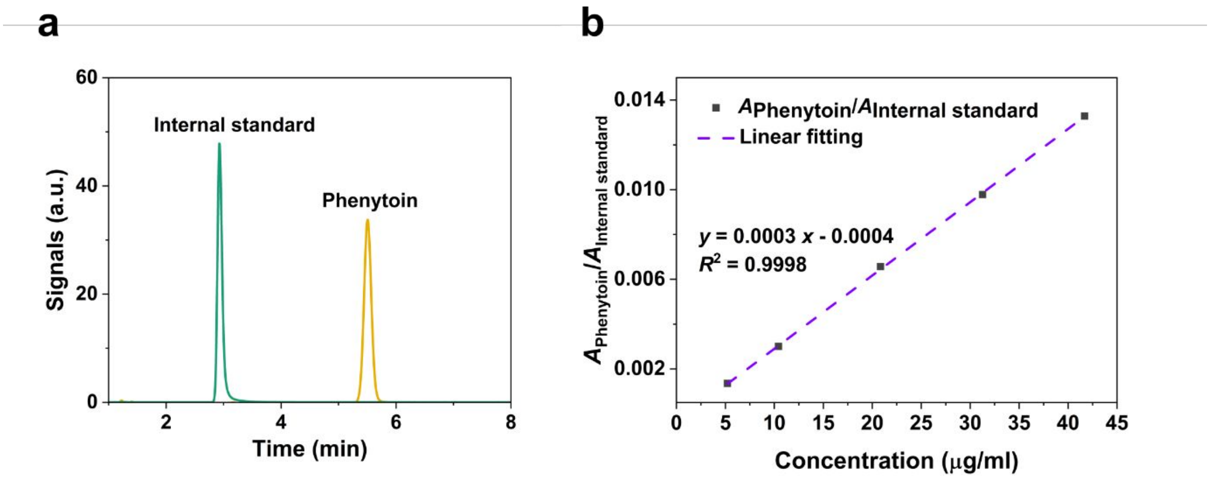

Figure S3. The calibration curve established with an internal standard. (a) HPLC chromatogram of phenytoin and the internal standard of 4-hydroxybenzonitrile. (b) Calibration curve of phenytoin-spiked serum samples in the range of 5-40 ug/mL. Six replicates are measured for each concentration. The standard deviation is too small to observe and within the size of the square symbols.

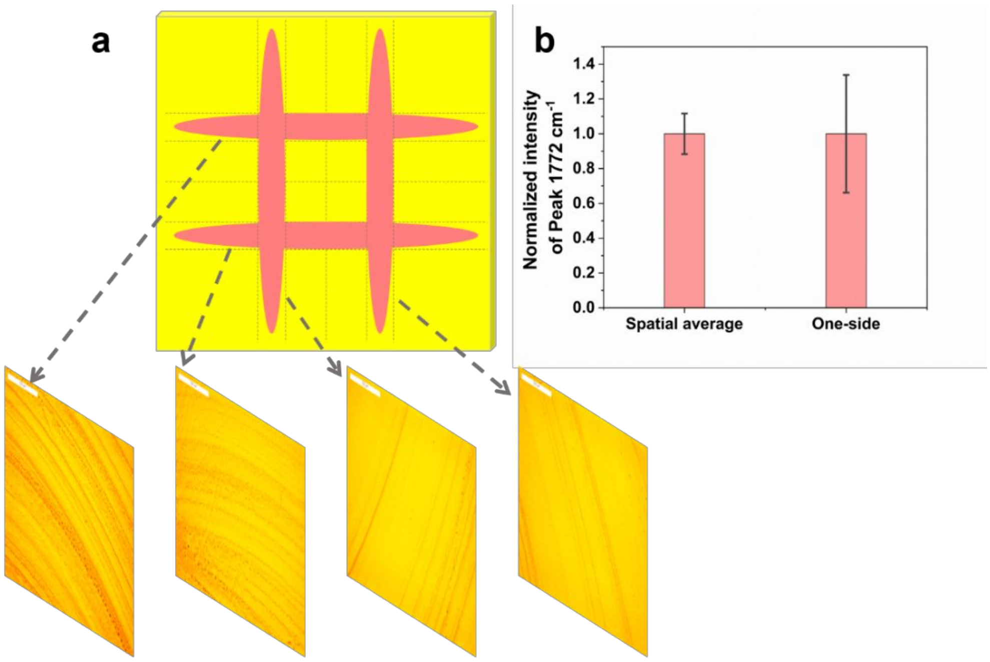

Figure S4. PM-IRRAS FTIR measurements with spatial averaging. (a) Optical microscopic images (bottom) of a dried spot of drug-spiked serum, taken from different areas of the sample substrate with four 90° rotations. The red areas in the schematic drawing (top) indicate the shape and position of the incident IR beam in the PM-IRRAS FITR on the substrate with a size of 25 mm by 25 mm; the beam is 3 mm above the centre of the substrate. (b) Standard deviations of the signals from a dried spot sample with 7 repeats were obtained using the methods of spatial averaging and one-side (no rotation) measurement. The standard deviation from the one-side method is approximately 3 times larger than the spatial averaging method.

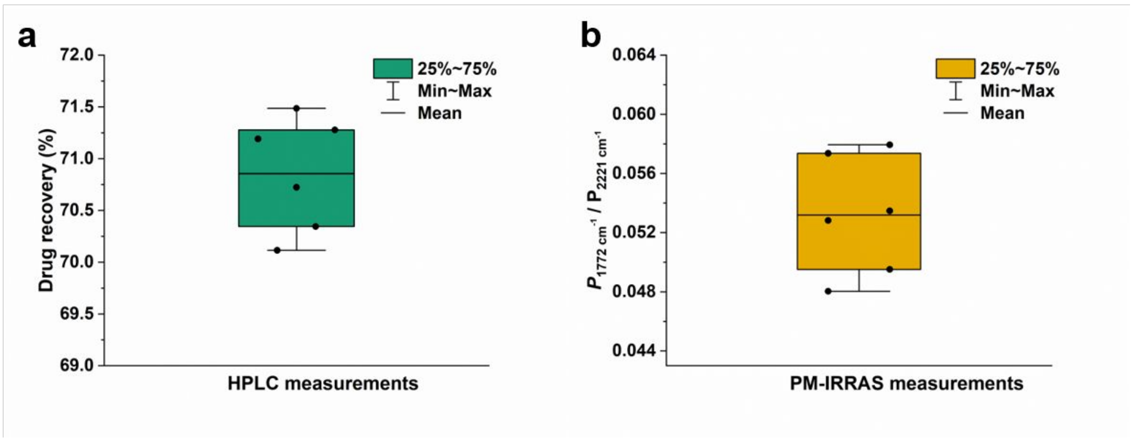

Figure S5. The relative standard deviations of the serum sample preparation protocol. (a) Drug recovery of 10 ug/mL phenytoin-spiked serum samples (*n*=6) following the sample preparation protocol, measured by HPLC. The relative standard deviation (RSD) is 0.8%, indicating minimal variation caused by the 3-step sample preparation process. (b) The ratio of the phenytoin target peak at 1772 cm^{-1} to the peak of 4-hydroxybenzonitrile (internal standard) at 2221 cm^{-1} for 10 ug/mL phenytoin-spiked serum samples (*n*=6) measured by PM-IRRAS. The RSD is 7.5%, showing it is acceptable to use PM-IRRAS for quantifying drug concentrations in human serum according to FDA`s Bioanalytical Method Validation Guidance for Industry.

Table 1 Costs of materials used in the established serum sample protocol.

**Materials**	**Unit Price in Market**	**Amount needed in the protocol**	**Cost per test (USD)**
Magnesium chloride hexahydrate	0.2 USD per gram	6.1×10^{-3} g	0.0012
Dextran sodium sulfate	4 USD per gram	6×10^{-4} g	0.0024
Ammonium sulfate	0.06 USD per gram	0.23 g	0.014
Ethyl acetate	0.05 USD per mL	2 mL	0.1
Gold	85 USD per gram	1.43×10^{-3} g	0.12
Silicon wafer	250 USD per square meter	6.25×10^{-4} m^2	0.16
		**Total**	0.40
